# PAR2 activation in the dura causes acute behavioral responses and priming to glyceryl trinitrate in a mouse migraine model

**DOI:** 10.1186/s10194-023-01574-5

**Published:** 2023-04-19

**Authors:** Bianca N. Mason, Shayne N. Hassler, Kathryn DeFea, Scott Boitano, Josef Vagner, Theodore J. Price, Greg Dussor

**Affiliations:** 1 Department of Neuroscience, University of Texas at Dallas, 800 West Campbell Rd, Richardson, TX 75080 USA; 2 Center for Advanced Pain Studies, University of Texas at Dallas, Richardson, TX 75080 USA; 3 PARmedics, Temecula, CA 92590 USA; 4 Department of Physiology, University of Arizona, Tucson, 85724 USA; 5 Bio5 Institute, University of Arizona, Tucson, AZ 85724 USA

**Keywords:** Migraine, Protease-activated receptor-2 (PAR2), Glyceryl trinitrate (GTN), 2at-LIGRL-NH_2_ (2AT)

## Abstract

**Background:**

Migraine is a severely debilitating disorder that affects millions of people worldwide. Studies have indicated that activation of protease-activated receptor-2 (PAR2) in the dura mater causes headache responses in preclinical models. It is also well known that vasodilators such as nitric oxide (NO) donors can trigger migraine attacks in migraine patients but not controls. In the current study we examined whether activation of PAR2 in the dura causes priming to the NO donor glyceryl trinitrate (GTN).

**Methods:**

A preclinical behavioral model of migraine was used where stimuli (PAR2 agonists: 2at-LIGRL-NH_2_ (2AT) or neutrophil elastase (NE); and IL-6) were applied to the mouse dura through an injection made at the intersection of the lamdoidal and sagittal sutures on the skull. Following dural injection, periorbital von Frey thresholds and facial grimace responses were measured until their return to baseline. GTN was then given by intraperitoneal injection and periorbital hypersensitivity and facial grimace responses observed until they returned to baseline.

**Results:**

We found that application of the selective PAR2 agonist 2at-LIGRL-NH_2_ (2AT) onto the dura causes headache-related behavioral responses in WT but not PAR2^−/−^ mice with no differences between sexes. Additionally, dural PAR2 activation with 2AT caused priming to GTN (1 mg/kg) at 14 days after primary dural stimulation. PAR2^−/−^ mice showed no priming to GTN. We also tested behavioral responses to the endogenous protease neutrophil elastase, which can cleave and activate PAR2. Dural neutrophil elastase caused both acute responses and priming to GTN in WT but not PAR2^−/−^ mice. Finally, we show that dural IL-6 causes acute responses and priming to GTN that is identical in WT and PAR2^−/−^ mice, indicating that IL-6 does not act through PAR2 in this model.

**Conclusions:**

These results indicate that PAR2 activation in the meninges can cause acute headache behavioral responses and priming to an NO donor, and support further exploration of PAR2 as a novel therapeutic target for migraine.

**Supplementary Information:**

The online version contains supplementary material available at 10.1186/s10194-023-01574-5.

## Background

Migraine is a debilitating headache disorder that negatively affects quality of life for millions of people worldwide [1]. Although migraine is highly prevalent, the underlying mechanisms that contribute to migraine are poorly understood. While clinical therapies are available, they often do not have the desired efficacy and/or result in unwanted side effects [2, 3]. Studies have suggested that abnormal activation of afferents from the trigeminal nerve that innervate the dura mater are integral in the generation of the headache phase of migraine attacks [4–8]. How these dural afferents become activated during a migraine is an important, yet still unanswered question. The potent vasodilator nitric oxide (NO) is a crucial modulator of meningeal vessels and is well known to trigger migraine attacks in people [9, 10]. NO donor infusion (such as with glyceryl trinitrate (GTN)) in migraineurs results in a migraine attack with a delay of 3 to 6 h [11, 12]. However, whether the role of nitric oxide in migraine involves vasodilation has been long debated and still remains inconclusive. Nitric oxide production and release can be stimulated by activation of several different migraine-relevant receptors found on meningeal vessels and neuronal fibers [13]. Given that nitric oxide is a key molecule in migraine pathophysiology, better understanding of the mechanisms that lead to modulation of NO sensitivity may lead to new therapeutic targets.

A well-positioned candidate to modulate NO sensitivity within the meninges is protease activated receptor 2 (PAR2) given its expression in mast cells [14] and nociceptors [15] and its role in other inflammatory disorders [16]. PAR2 has been demonstrated to promote meningeal vasodilation through an NO-dependent mechanism [17], and promote pain transmission through sensitization of peripheral nociceptors [18]. PAR2 is a Ca^2+^ mobilizing G-protein coupled receptor (GPCR) from the PAR [1–4] family [19, 20]. These receptors are unique because their mechanism of activation is through a “tethered ligand” that is created following cleavage by proteases. Typical proteolytic cleavage of an N-terminal extracellular site on PAR2 via trypsins, tryptases, and kallikreins is necessary for PAR2 to interact with its tethered ligand, activate G proteins, and increase intracellular calcium. PAR2 is then internalized via endocytosis and re-expression requires replacement with a functional PAR receptor [19, 20]. A growing number of endogenous proteases such as neutrophil elastase can also cleave PAR2 [21] and activate PAR2 signaling via distinct signaling mechanisms. It has been reported that neutrophil elastase induces cAMP accumulation, ERK1/2 activation, and transient receptor potential vanilloid (TRP) channel activation in a PAR2-dependent manner in mouse nociceptors [22]. However, in contrast to trypsin-activated PAR2 signaling, neutrophil elastase may activate PAR2 via a mechanism that does not trigger exposure of a tethered ligand domain, β-arrestin recruitment, or receptor internalization [21]. Additionally, synthetic peptidomimetic activating peptides such as SLIGRL-NH_2_ or SLIGVK-NH_2_ can also directly activate PAR2 by mimicking the canonically-exposed tethered ligand domain of PAR2. It is not clear whether functional differences exist following canonical activation compared to other modes of activation such as with neutrophil elastase.

We have shown induction of canonical PAR2 signaling with the highly selective PAR2 agonist 2at-LIGRL-NH_2_ (2AT) causes migraine-like pain behaviors (cutaneous mechanical hypersensitivity and grimace) when administered onto the dura of mice [23]. These behaviors were attenuated by administration of the migraine drug sumatriptan and by PAR2 selective antagonist C391. In addition to the in vivo studies, our in vitro studies showed that 2AT induced Ca^2+^ signaling in both neuronal and non-neuronal cells and this was also inhibited by C391 [15, 23]. It has been reported that PAR2 activation leads to vasodilation of dural arteries in rodents and this in part is mediated by modulation of NO [17, 24]. PAR2 has also been shown to increase NO production in multiple cell types via activation of NO synthase [24–26]. We have previously shown that PAR2 both promotes prostaglandin E2 (PGE2) production and increases sensitivity to PGE2 associated with transition to the chronic pain state [27]. We proposed, therefore, that activation of PAR2 might also increase sensitivity to NO. Given the potential relationship between NO and PAR2 in migraine, we aimed to establish whether PAR2 activation plays a significant role in NO-mediated behavioral responses in a preclinical model of migraine.

In the studies described here, we show that PAR2 activation in the dura of wild-type mice induces migraine-like behaviors and hyperalgesic priming to GTN, both of which are attenuated in global PAR2 knockout (PAR2KO) mice. Importantly, we demonstrate a role for PAR2 in response to dural stimulation with both a selective agonist and neutrophil elastase, each activating the receptor via distinct mechanisms. These data add to the building literature supporting PAR2 as a potential therapeutic target for migraine.

## Materials and methods

### Animals

Two strains of wild-type mice were used: ICR mice (Envigo; Indianapolis, IN) were used in sumatriptan experiments, and wild type C57BL/6 J and PAR2KO mice in a C57BL6J background (Jackson Laboratories; Bar Harbor, ME) were used in all other studies. Both adult male and female mice between 5- to 8-week old were used in the experiments. All mice were bred in-house at University of Texas at Dallas and housed in groups of 3–5 per cage in a climate-controlled room with a 12-h light/dark cycle with food and water ad libitum. For all experiments, investigators were blinded to genotype and drug treatments. All experiments and procedures were performed in accordance with the guidelines recommended by the National Institute of Health and the International Association for the study of Pain. Experiments and procedures were additionally approved by the Institutional Animal Care and Use Committee at University of Texas at Dallas.

### Dural and intraperitoneal drug administration

All drugs that required dilution were prepared with either synthetic interstitial fluid (SIF; dural injections) or PBS (Fisher Scientific; intraperitoneal (i.p.) injections). For dural injections the doses were given as follows: 5 μl of SIF with less than 1% DMSO used as vehicle; 2 pmol/μl (1.395 ng/μl) for a total of 10 pmol (6.975 ng) of 2at-LIGRL-NH_2_ (2AT); 2 units/μl for a total of 10 units of human sputum neutrophil elastase (Elastin Products Company; Owensville, MO); 0.02 ng/μl for a total of 0.1 ng of rat recombinant IL-6 (R&D Systems; Minneapolis, MN). For intraperitoneal injections the doses were given as follows: 0.6 mg/kg of sumatriptan (Tocris; Bristol, UK) or 1 mg/kg of glyceryl trinitrate (GTN; Sigma; St. Louis, MO) unless otherwise indicated. For all i.p. injections, sumatriptan, GTN, or PBS (vehicle) were administered at 10 μl /g bodyweight with a 30 g × 0.5 in needle. All injections were performed by either B.N.M. or S.N.H. Doses of PAR2 agonists, 2AT, sumatriptan, NE and IL6 were chosen based on previous studies [15, 23, 28]. Pilot experiments (not shown) determined that 1 mg/kg GTN, but not higher doses, did not produce behavioral responses in the absence of pretreatment with 2AT, NE or IL-6.

**Fig. 1:**
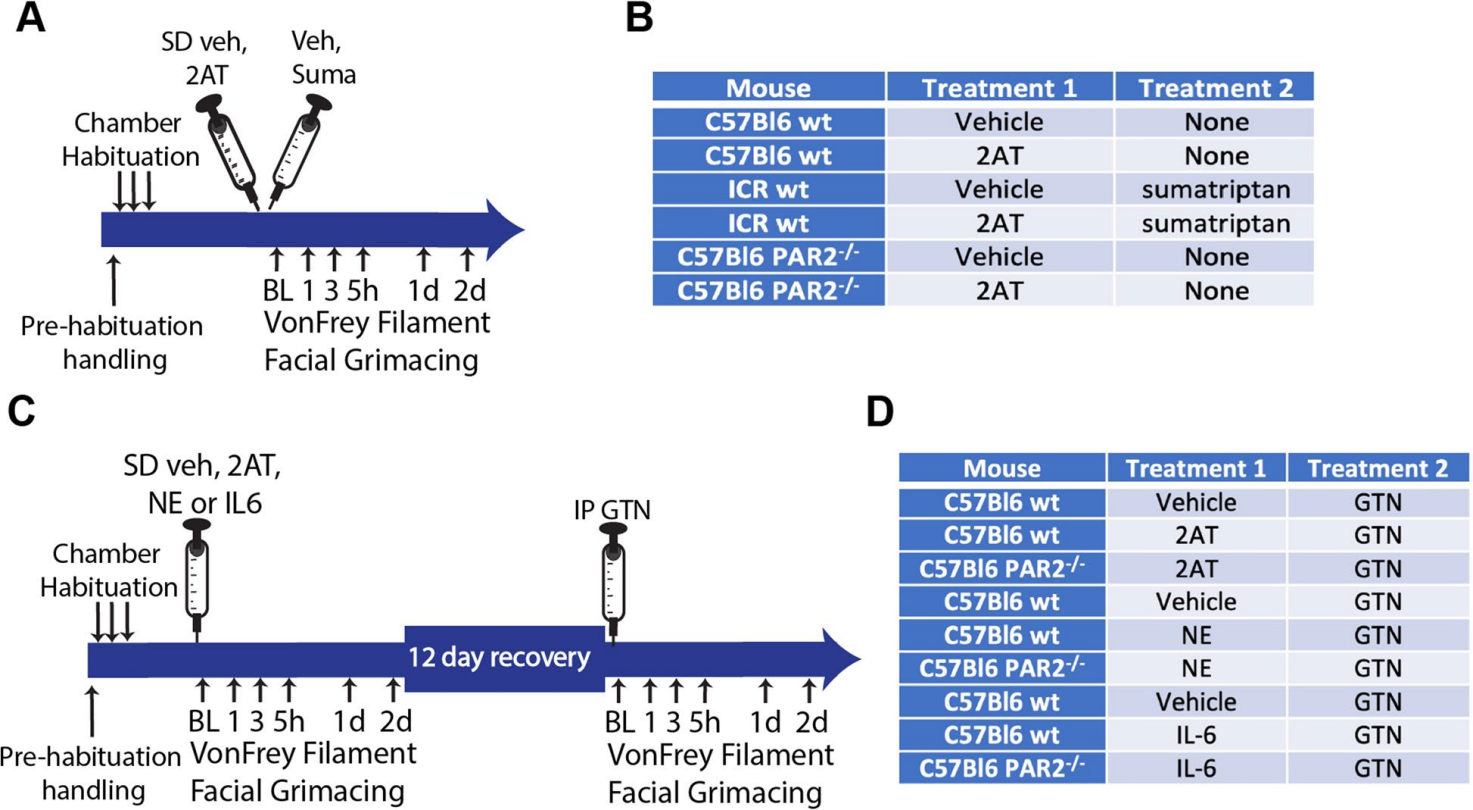
*Injection and testing protocol and treatment groups.* Descriptions of the time course shown in A and C are found in the methods. Panels A and B refer to the data in Fig. 2. Panels C and D refer to the data in Figs. 3, 4, and 5. Abbreviations not found at the end of the text: Supradural (SD), sumatriptan (suma), baseline (BL)

The experimental design for acute and chronic mouse migraine models are diagramed in Fig. 1. Mouse dural injections were performed as previously characterized [29] and a video article that describes the procedure in its entirety is available [30]. On Day 1, mice were anesthetized using isoflurane for approximately 2 min and injected with vehicle, 2AT, neutrophil elastase, or IL-6 onto the dura using a modified internal cannula (Invivo1, part 8IC313ISPCXC, Internal Cannula, standard, 28 gauge). A plastic guide was used to maintain the inner length of the cannula to 0.5 to 0.65 mm in length based on the weight of the mouse to ensure the dura was not punctured. The dorsal cranium was carefully injected at the intersection of the sagittal and lambdoidal sutures with the plastic guide preventing damage of the underlying dura. A solution volume of 5 μl was injected onto the dura. The animals were removed from the anesthesia and placed in the testing chamber for behavioral testing. Mice are returned to their cages and to the animal facility until testing at later time points, 24 and 48 h later. For GTN sensitization assays, baseline behavioral measurements were obtained on day 14 and mice were administered an intraperitoneal injection of GTN, placed in the chambers and behavioral assays performed at the indicated time points. Mice are returned to the animal facility until testing at later time points, 24 and 48 h later.

### Mechanical hypersensitivity and grimace assays

#### Facial hypersensitivity

Mice were handled for a single 5-min session approximately 24 h prior to habituation to the behavior chambers. Following handling, mice were habituated to the chamber for 2 h/day for 3 consecutive days in 4 oz paper cups (Choice Paper Company; Brooklyn, NY) with a pellet of chow as previously characterized [29]. Facial mechanical sensitivity baselines were assessed by applying von Frey filaments to the periorbital region of the face (midline of the forehand) prior to drug administration. Baselined mice were defined as mice that exhibited a withdrawal threshold between 0.5 g – 0.6 g. Mice that displayed a threshold of < 0.5 g were not used in this study. Additionally, filaments > 0.6 g were also not used on mice in this group of experiments. Following injections, von Frey filaments were applied to the periorbital region using the Dixon up-down method. A response was defined as swiping the filament away from the face during von Frey application. A “no response” is indicated by periorbital application of the von Frey filament for 3–5 s without withdrawal. During the testing period, responses were recorded at 1, 3, 5, 24, and 48 h following drug treatments. All testing was conducted by investigators blinded to the experimental groups.

#### Mouse grimace scale

The protocol originally developed by Mogil and colleagues for testing facial grimacing in mice was utilized for this study [31]. Mice were placed individually in transparent, acrylic testing chambers (4 × 3x3.5 in). Two high-resolution (1920 × 1080) digital video cameras (High-definition Handycam Camcorder, model HDR-CX100, Sony, San Jose, CA) were placed immediately outside both acrylic glass walls to maximize the opportunity for clear head shots. The animals were then recorded for 20 min and the photographs that included views of the mouse face were extracted from each recording and scored by blinded scorers. The scores were averaged at each time-point by treatment group. All behavioral assays were performed by either B.N.M. or S.N.H.

### Statistical analysis

Each experiment was conducted separately with a new cohort of mice. In most experiments a two-way, repeated measures ANOVA (factors: time, treatment) were used. Bonferroni multiple-comparisons test was used as the post hoc analysis. In each graph, each group was compared against every other group. Data were analyzed using Graphpad Prism 9, version 9.0.0 software. Significance was set at *p* < 0.05 for all analyses. G power was used to generate a power analysis with expected effected size based on calculations reported in Avona et al., 2021 [32]. All sample sizes for periorbital von Frey were greater than or equal to the suggested sample size of 4 calculated in G power for a 0.8 desired power. The family of tests used in G power was F tests and statistical test was repeated measures ANOVA (between factors). The type of power analysis was A priori with a treatment effect size of 36.04, alpha of 0.05, and the number of measurements used was 7. All data are graphed as means ± SEM unless otherwise noted.

## Results

### Loss of PAR2 inhibits 2AT-induced hypersensitivity in both male and female mice

**Fig. 2:**
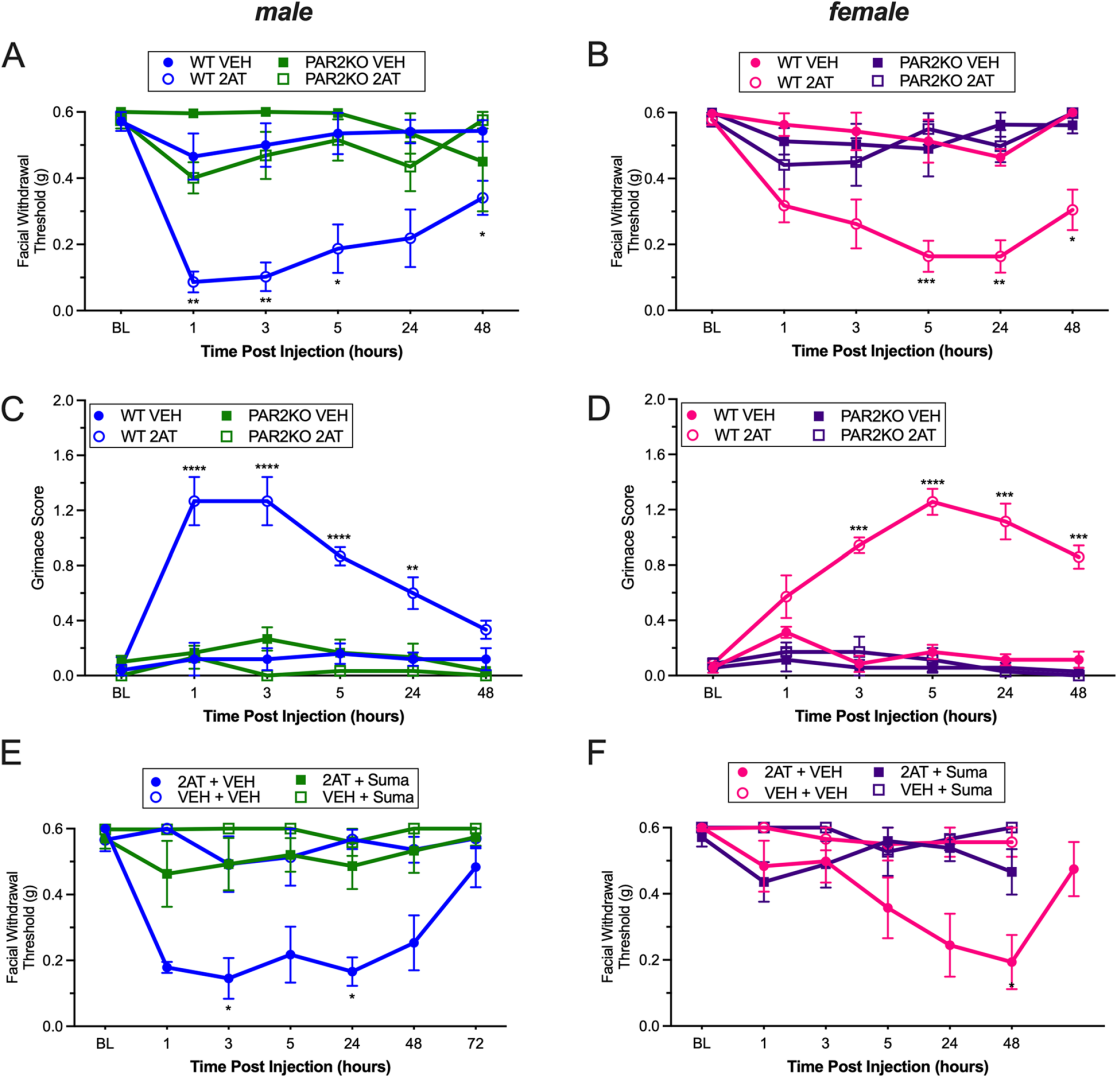
*Global loss of PAR2 and administration of sumatriptan inhibits dural 2AT-induced hypersensitivity*. **A** Facial withdrawal thresholds in male PAR2KO mice and their WT controls following treatment with either 2AT or vehicle. WT male mice administered 2AT (*n* = 7) exhibited significantly decreased withdrawal thresholds compared PAR2KO mice treated with 2AT (*n* = 7) and both WT (*n* = 4) and PAR2KO (*n* = 6) mice treated with vehicle. **B** Facial withdrawal thresholds in female PAR2KO mice and their WT controls following treatment with either 2AT or vehicle. WT female mice administered 2AT (*n* = 7) exhibited significantly decreased withdrawal thresholds compared PAR2KO mice treated with 2AT (*n* = 8) and both WT (*n* = 7) and PAR2KO (*n* = 6) mice treated with vehicle. **C** Grimace scores of male PAR2KO mice and their WT controls following dural application of either 2-AT or vehicle. WT male mice administered 2AT exhibited significantly increased grimacing compared to PAR2KO mice treated with 2AT and both WT and PAR2KO mice treated with vehicle. **D** Grimace scores of female PAR2KO mice and their WT controls following dural application of either 2AT or vehicle. WT female mice administered 2AT exhibited significantly increased grimacing compared to PAR2KO mice treated with 2AT and both WT and PAR2KO mice treated with vehicle. **E** Facial withdrawal thresholds in male WT mice administered 2AT and sumatriptan. Sumatriptan significantly attenuates withdrawal thresholds induced by 2AT (2AT + Suma; *n* = 5) compared to 2AT + Veh (*n* = 6) **F** Facial withdrawal thresholds in female WT mice administered 2AT and sumatriptan. Sumatriptan significantly attenuates withdrawal thresholds induced by 2AT (2AT + Suma; *n* = 7) compared to 2AT + Veh (*n* = 7). *p < 0.05, **p < 0.01, ***p < 0.001, ****p < 0.0001. Data are represented as mean ± SEM

We have reported that 2AT is highly selective at the PAR2 receptor in vitro and in vivo [23, 33, 34]. To assess the selectivity of 2AT in this preclinical migraine model, we evaluated the effect of dural 2AT in global PAR2KO mice on a C57BL6 background. Following dural injection of 2AT (10 pmol), both male and female PARKO mice failed to exhibit a hypersensitive phenotype compared to age-matched WT mice treated with 2AT (Fig. 2A; F_(3,20)_ = 19.51, *p* < 0.0001, 2B; F_(3,24)_ = 32.39, *p* < 0.0001). The observed hypersensitive profile was indistinguishable from both male and female WT and PAR2KO mice treated with vehicle. Moreover, the affective component of pain was also assessed using the mouse grimace scale. Male WT mice treated with 2AT had a significant increase in grimace scores at 1, 3, 5, and 24 h post injection whereas there was no grimace detected in either vehicle-treated mice nor 2AT-treated PAR2KO mice (Fig. 2C; F_(3,16)_ = 33.82, *p* < 0.0001). Female WT mice had a significant increase in grimace scores at the 3, 5, 24, and 48 h timepoints following 2AT, with no effect in PAR2KO mice and no effect of vehicle (Fig. 2D; F_(3,24)_ = 98.65, *p* < 0.0001). There was no significant difference in grimace profile detected between male and female PAR2KO mice (F_(1,8)=_0.53; *p* = 0.487). We then compared the responses of 2AT in male (Fig. 2A) and female (Fig. 2B) WT mice. Overall, sex does not significantly affect the behaviorial responses to 2AT (F_(1,13)_ = 1.61, *p* = 0.2255). A significant difference in facial withdrawal threshold between the sexes was only detected at the 1 h timepoint (*p* = 0.0110) (Fig. S1A,C). The grimace score showed a significant difference at 1, 5 and 24 h timepoints (Fig. S1B); however, this reflected a difference in the time to maximum response. The mean grimace score over the 1–24 h time period was not significantly different between the two groups (Fig. SIC, *p* = 0.183). These data confirm the selectivity of 2AT and show a lack of sex differences in the ability of 2AT to induce significant behavioral responses in this model.

We next tested whether 2AT responses in male and female ICR mice are attenuated by the standard acute migraine therapeutic sumatriptan. While we have shown efficacy of sumatriptan against dural 2AT responses in males [23], it was unclear whether similar efficacy is observed in females. Mice were given a dural injection of either 2AT (10 pmol) or vehicle. Immediately after dural application, mice were then given an intraperitoneal injection of 0.6 mg/kg sumatriptan or vehicle approximately 1 h prior to testing for facial hypersensitivity. Similar to our previous reports in C57BL6 mice [23], the dural 2AT induced facial hypersensitivity and this was significantly attenuated by sumatriptan in males (Fig. 2E; F_(3,18)_ = 30.98,* p* < 0.0001). In female ICR mice, while the hypersensitivity time course was slightly different than males, with dural 2AT-induced hypersensitivity significant at the 24 and 48 h timepoints, there was a similar significant block of the response by sumatriptan (Fig. 2F; F_(3,19)=_3.00, *p* = 0.056). There was no significant difference in the mean responses of male and female mice (Fig. S2). These data show that dural 2AT produces sumatriptan-sensitive behavioral responses through selective activation of PAR2 and that there are no sex differences in the effects. Given the lack of sex differences in the overall responses, the remainder of the studies were conducted in male C57BL6 mice.

### Acute PAR2 activation is sufficient to enhance hyperalgesic priming to GTN

**Fig. 3:**
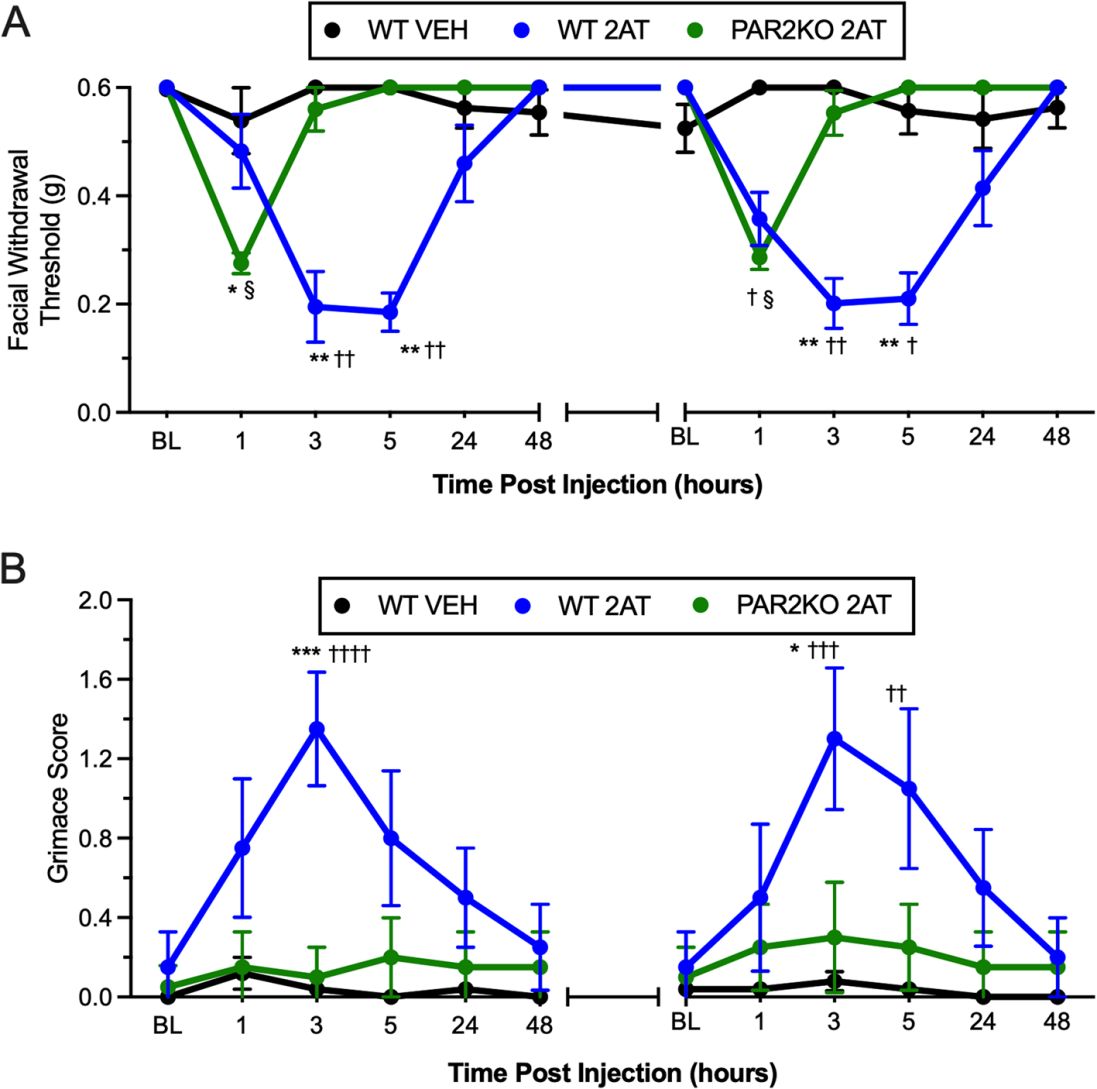
*Dural 2AT causes hyperalgesic priming to GTN*. **A** Facial withdrawal thresholds in PAR2KO mice (*n* = 4) following treatment of 2AT, WT controls following treatment of 2AT (*n* = 4), and WT controls (*n* = 4) following administration of vehicle and subthreshold GTN. Note that there are 12 days between the last time point of 48 h on the left and the baseline for GTN administration, indicated by the broken x-axis (see Fig. 1C). Application of 2AT significantly decreased withdrawal thresholds in WT mice and enhanced hyperalgesic priming to GTN compared to WT mice treated with vehicle (†) and PAR2KO mice treated with 2AT (*). PAR2KO mice previously treated with 2AT were hypersensitive at 1 h following dural 2AT and GTN administration compared to vehicle treated mice (§). **B** Grimace scores of PAR2KO mice and their WT controls. 2AT significantly increased grimacing in the acute phase in WT mice. *,^†^*p* < 0.05, **,^††^*p* < 0.01, ***,^†††^*p* < 0.001, ^††††^*p* < 0.0001. Data are represented as mean ± SEM

Since NO is highly implicated in the pathophysiology of migraine, we next tested whether activation of PAR2 on the dura was able to cause hyperalgesic priming to subthreshold doses of the NO donor GTN. Either vehicle or 2AT (10 pmol) was applied to the dura of both PAR2KO and WT mice. After the initial hypersensitivity had resolved (approximately 14 days following injection), mice were administered 1 mg/kg GTN and assessed for hyperalgesic priming. In vehicle treated WT mice, no significant difference in facial withdrawal threshold or facial grimacing, compared to baseline, was observed with 1 mg/kg GTN (Fig. 3, black circles; *p* > 0.999). 2AT significantly decreased facial withdrawal thresholds, compared to vehicle controls, in WT mice at the 3 and 5 h timepoints, and administration of subthreshold GTN 14 days later resulted in a similar decrease in facial withdrawal at 3–5 h (Fig. 3A blue circles; *p* < 0.001) that was absent in PAR2KO mice treated with 2AT (Fig. 3A green circles, *p* > 0.999). There was no significant difference in withdrawal threshold observed between 2AT-treated PAR2KO mice and vehicle-treated WT mice (Fig. 3A; F_(2,9)_ = 129.00, *p* < 0.0001). There was an acute effect of both dural 2AT injection and intraperitoneal GTN injection in the PAR2KO mice, but neither lasted beyond the 1-h post-injection time point. It is unclear in the initial phase whether this is a response in PAR2KO mice to dural injection itself as responses have been observed at this early time point due to the injection procedure. The response at the 1 h time point following GTN in PAR2KO mice may simply reflect a hypersensitivity to injection in these mice given that this effect was transient. Following administration of 2AT, grimace scores were also increased in WT mice treated with 2AT compared to mice treated with vehicle and PAR2KO mice treated with 2AT (Fig. 3B; F_(2,120)_ = 33.45, *p* < 0.0001). Similarly, GTN administration in WT, but not PAR2KO mice that were previously treated with 2AT displayed significant increases in grimace scores. These data suggest that canonical activation of PAR2 via dural application of 2AT is sufficient to enhance priming to a subthreshold dose of the NO donor GTN and may contribute to the initiation and maintenance of migraine-like behavior.

### Loss of PAR2 attenuates acute neutrophil elastase-induced pain behaviors and hyperalgesic priming to GTN

**Fig. 4:**
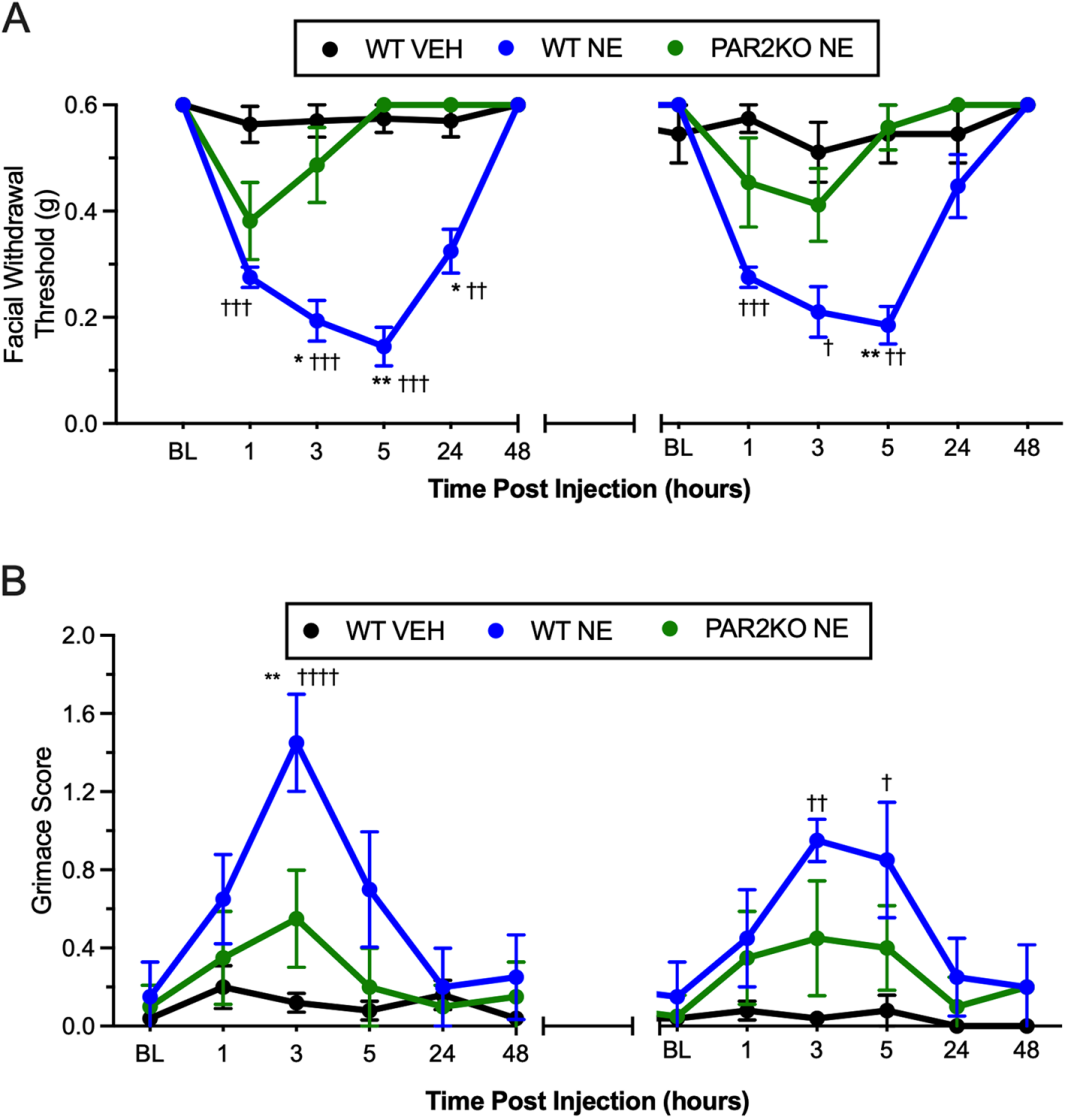
*Dural neutrophil elastase (NE) causes hyperalgesic priming to GTN.* **A** Facial withdrawal thresholds in PAR2KO mice following treatment of NE (*n* = 4), WT controls following treatment of NE (*n* = 4), and WT controls following administration of vehicle (*n* = 5) and subthreshold GTN. Note that there are 12 days between the last time point of 48 h on the left and the baseline for GTN administration, indicated by the broken x-axis (see Fig. 1C). Application of NE significantly decreased withdrawal thresholds in WT mice and enhanced hyperalgesic priming to GTN compared to WT mice treated with vehicle (†) and PAR2KO administered NE (*). **B** Grimace scores of PAR2KO mice and their WT controls. NE significantly increased grimacing in WT mice compared to WT mice treated with NE. *, ^†^*p* < 0.05, **,^††^*p* < 0.01, ^†††^*p* < 0.001, ^††††^*p* < 0.0001. Data are represented as mean ± SEM

Our prior study showed that responses to dural mast cell degranulation were lost in global PAR2KO mice [23]. Neutrophil elastase is also a known activator of PAR2 but whether dural application of neutrophil elastase causes behavioral responses in this preclinical migraine model is not known. Unlike the direct agonist 2AT, neutrophil elastase activates PAR2 signaling by cleaving the N-terminal sequence and selectively activating MAPK signaling via a G_12/13_/Rho-dependent pathway [21]. Therefore, we determined whether dural application of neutrophil elastase could also cause behavioral responses and priming to GTN that are dependent on PAR2. Following dural application of 10 units of neutrophil elastase, WT mice exhibited a significant decrease in their facial withdrawal threshold at 1, 3, 5, and 24 h compared to vehicle-treated mice (Fig. 4A; F_(2,10)_ = 70.20, *p* < 0.0001). PAR2KO mice treated with neutrophil elastase had significantly higher withdrawal thresholds than WT mice treated with neutrophil elastase at the 3, 5, and 24 h timepoints. We observed a decrease in facial withdrawal threshold at 1 h in PAR2KO mice that was not significantly different from wt mice, possibly reflecting a response to the dural injection itself in the PAR2KO. Similar to 2AT, neutrophil elastase caused priming to GTN in WT mice, but not PAR2KO, with significant hypersensitivity at 1, 3, and 5 h after administration. WT mice treated with neutrophil elastase also exhibited an increase in grimace 3 h after injection compared to PAR2KO mice treated with neutrophil elastase and vehicle-treated mice (Fig. 4B; F_(2,120)=_23.30, *p* < 0.0001). We did not detect any significant difference in grimace following GTN administration between WT and PAR2KO mice treated with neutrophil elastase. However, WT mice previously treated with neutrophil elastase grimaced significantly more than those mice previously treated with vehicle, while there was no significant difference from vehicle in the PAR2KO group. These data show that activation of PAR2 via non-canonical mechanisms and subsequent signaling cascades is sufficient to induce migraine-like pain behaviors and hyperalgesic priming to GTN.

### IL-6 increases sensitivity to GTN through a PAR2-independent mechanism

**Fig. 5:**
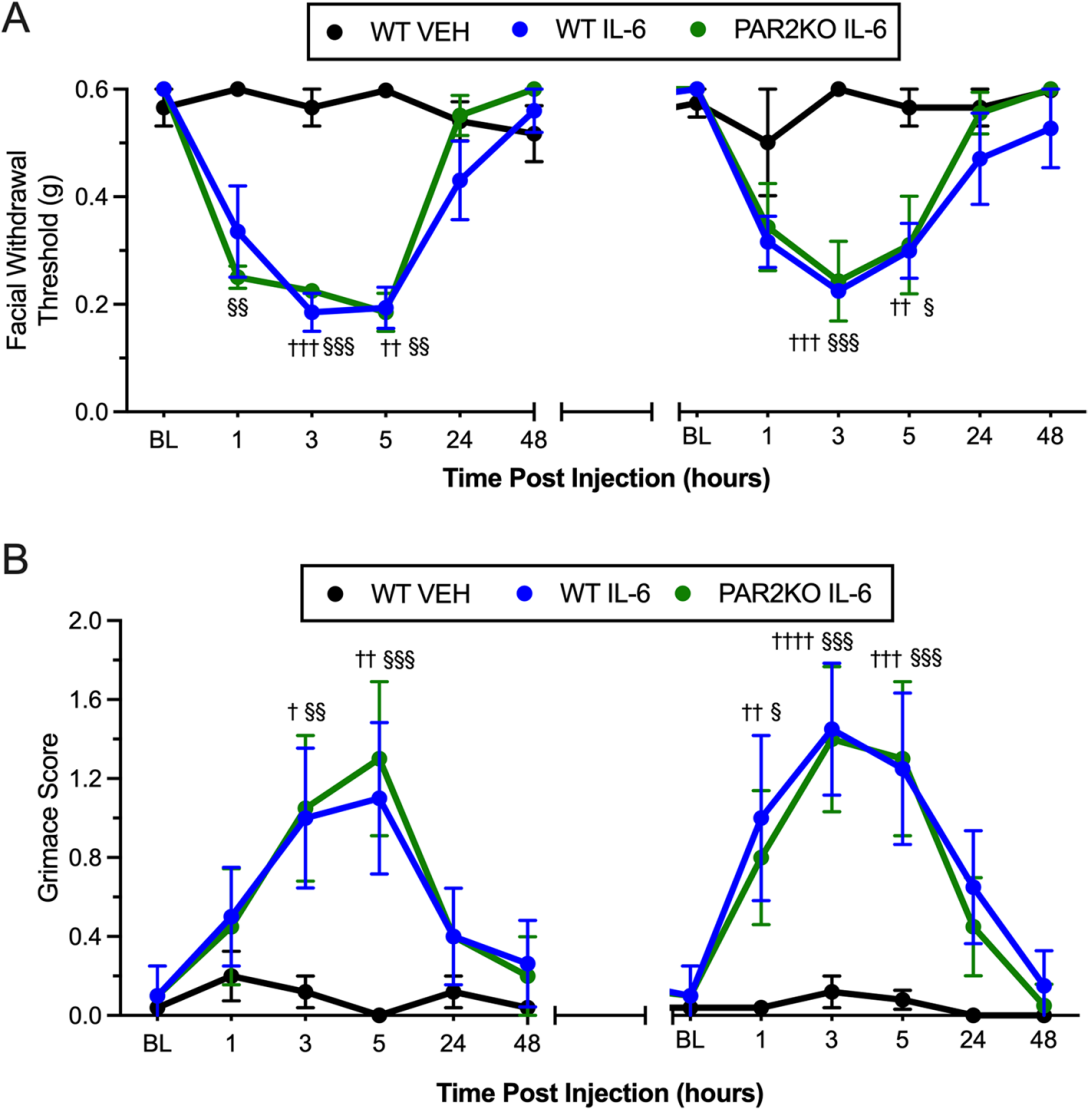
*Dural IL-6 causes hyperalgesic priming to GTN in the absence of PAR2*. **A** Facial withdrawal thresholds in PAR2KO mice following treatment with IL-6 (*n* = 4), WT controls (*n* = 4) following treatment of IL-6, and WT controls following treatment with vehicle and subthreshold GTN. Note that there are 12 days between the last time point of 48 h on the left and the baseline for GTN administration, indicated by the broken x-axis (see Fig. 1C). Application of IL-6 significantly decreased withdrawal thresholds in both WT (†) and PAR2KO (§) mice and enhanced hyperalgesic priming to GTN compared to WT mice treated with vehicle. **B** Grimace scores of PAR2KO mice and their WT controls following treatment with IL-6 and GTN. IL-6 significantly increased grimacing in WT and PAR2KO mice compared to WT mice treated with vehicle. There is no significant difference in grimacing between Par2KO and WT mice treated with IL-6. ^†,§^*p* < 0.05, ^††,§§^*p* < 0.01, ^†††,§§§^*p* < 0.001, ^††††^*p* < 0.0001. Data are represented as mean ± SEM

Interleukin-6 (IL-6) is a pro-inflammatory cytokine that is found to be elevated during migraine attacks. We have shown that IL-6 causes sensitization of meningeal nociceptors and behavioral responses following dural application in rodents [28]. IL-6 has been shown to induce significant release of neutrophil elastase from cultured neutrophils [35]. Given that 2AT and neutrophil elastase both modulated NO responses through a PAR2 mechanism, we determined whether IL-6 responses were also mediated through downstream activation of PAR2. WT and PAR2KO mice were given a dural injection of 0.1 ng of IL-6 or vehicle. IL-6 elicited a robust decrease in facial withdrawal threshold in both WT and PAR2KO mice compared to vehicle-treated mice (Fig. 5A; F_(2,10)_ = 74.34, *p* < 0.0001). Similar to the acute phase, GTN also caused a decrease in facial withdrawal thresholds in both WT and PAR2KO mice that were previously treated with IL-6 at 3 and 5 h following injection. IL-6 elicited a marked increase in grimace scores in both WT and PAR2KO mice compared to mice treated with vehicle. GTN also caused increased grimacing but there was no genotype effect observed between WT and PAR2KO mice previously treated with IL-6 (Fig. 5B; F_(2,120)_ = 29.71, *p* < 0.0001). These data suggest that IL-6 and PAR2 sensitize meningeal afferents and enhance sensitivity to GTN by distinct mechanisms in this model.

## Discussion

PAR2 has been strongly implicated in a variety of chronic pain disorders [20] and an emerging role for PAR2 has been shown in migraine models. Previous studies in rodents using in vivo electrophysiology show that PAR2 activation sensitizes meningeal nociceptors [18]. Additionally, we have reported that PAR2 activation is a key mechanism by which mast cell degranulation in the dura causes acute behavioral responses (facial withdrawal, paw withdrawal and facial grimacing) in mice, that are inhibited by IP injection of sumatriptan or a PAR2 antagonist in males [23]. To build on these findings in this study, we confirmed that our prior findings in males are also observed in females. Additionally, we measured both mechanical responses and affective dimensions of pain in response to another endogenous activator of PAR2 (neutrophil elastase) in wild type and PAR2 knockout mice, and examined the role of PAR2 in the development of sensitivity to a nitric oxide donor, a common migraine trigger. We show that dural application of both a selective PAR2 agonist and neutrophil elastase are sufficient to elicit a migraine-like behavioral response in mice. While a decrease in facial withdrawal threshold was observed at 1 h after both 2AT and NE administration in PAR2KO mice, it is unclear whether this was due to a PAR2-independent component to the acute response or inconsistency often observed at this time point, as transient behavioral changes can occur due to the supradural injection itself. We also show that this acute response is followed by hyperalgesic priming to a non-noxious dose of GTN, administered 12 days after the acute phase had resolved (i.e. day 14 post NE injection). The acute hypersensitivity and grimace behaviors in response to PAR2 activation were similar between sexes, and sumatriptan is sufficient to attenuate this phenotype in both males and females. Our findings suggest that actions of IL-6 are likely not due to downstream activation of PAR2 given that neither IL-6 responses nor priming to GTN were affected by loss of PAR2. Taken together, our present findings support the idea that PAR2 activation can increase sensitivity to NO leading to a pain phenotype similar to that observed in migraine patients and further support that antagonism of PAR2 may be a novel therapeutic approach for migraine.

Sensitivity to NO donors is a hallmark of migraine pathophysiology [9, 36] but the underlying mechanisms by which NO contributes to migraine are not clear. Previous studies have investigated a link between PAR2 and migraine, and have suggested PAR2 activation results in direct sensitization of meningeal nociceptors [18] and dilation of meningeal vessels mediated in part through production of NO [24]. We have shown that 2AT can induce hyperalgesic priming to an innocuous dose of PGE2 in the paw via a mechanism involving MAPK-dependent protein translation and postsynaptic sensitization [27].To our knowledge no other studies have examined the ability of PAR2 to cause a similar sensitization to NO. Here we show that dural PAR2 activation via 2AT and neutrophil elastase is sufficient to enhance responses to intraperitoneal administration of GTN and this is lost in PAR2KO mice. PAR2 activation causes granulocyte infiltration [37] in inflammatory pain models, hence, it is possible that PAR2 activation promotes mast cell and/or neutrophil infiltration and subsequent activation to initiate nociceptor plasticity in this model. In addition to vasculature and sensory neurons, glial cells and immune cells also express PAR2 [38] and there is a rich network of immune cells residing in the dura [39]. Whether enhancement of NO sensitivity by PAR2 activation requires a specific cell type in the dura is an important question to address in future studies, as well as whether PAR2-induced protein translation is required. Although PAR2 causes priming to GTN, whether PAR2 activation can induce priming to other subthreshold migraine-relevant stimuli is not yet known.

There has been an increase in research regarding sex differences in pain models [40–42]. To date, there have been no studies that report a difference in pain phenotype following PAR2 activation. Here, we show that activation of PAR2 by the agonist 2AT did not cause a significant difference in behavioral responses between male and female mice. Considering that this mode of activation of PAR2 does not induce sex differences in responses, it seems unlikely that activation of PAR2 other canonical modes such as via tryptase or non-canonical elastase cleavage of the N-terminus would yield a difference in pain phenotypes between sexes. Similarly, we have only shown that IL-6 modulates responses to NO through a PAR2-independent mechanism in males, but we have previously reported that IL-6 administration onto the dura elicits pain behaviors equally between sexes. Therefore, we do not expect that sex differences in the response to IL-6 would be present in this study. We did observe that the temporal response patterns were slightly different between males and females following dural application of 2AT. Females had a greater delay in the onset of pain behaviors compared to males but there was no difference in the intensity of the response observed. The reason for this phenotype is unknown. However, it may reflect a sex difference in PAR2 expression in the dura mater. Although there are no studies that directly compare PAR2 expression between males and females in the dura or trigeminal ganglion, PAR2 expression has been reported higher in males in other cell populations [43]. While it is unlikely that major sex differences exist once membrane PAR2 is activated, there could be sex differences in the upstream events that lead to PAR2 activation, especially immune cell-based events where examples of sex differences are continuing to emerge. The findings reported here bypass the activation of immune cells by using 2AT or neutrophil elastase but it is possible that mechanisms exist where immune cell activity might lead to differential activation of PAR2 between the sexes. Dissecting the upstream events leading to differential activation of PAR2 between sexes may prove useful for understanding the role of PAR2 in migraine.

Migraine is associated with high levels of IL-6 in some patients [44, 45] and high levels of IL-6 in humans results in neutrophil degranulation and subsequent release of neutrophil elastase [35]. Therefore, it is a reasonable assumption that PAR2 activation may potentially be a downstream consequence of IL-6 receptor activation on neutrophils. However, loss of PAR2 did not affect the pain behaviors induced by IL-6 or the priming response to GTN. Macrophages and mast cells make up approximately 63% of immune cells in the dura while neutrophils only represent 5 percent [39]. The low number of neutrophils in the dura may be an explanation as to why loss of PAR2 did not alter the IL-6 response. Although elastase can be released from macrophages [46], mast cells [47], endothelial cells [48], and even smooth muscle [49], it is not known whether IL-6 can cause elastase release from either of these cell types. While PAR2 activation can induce release of inflammatory cytokines including IL-6 [50–52], our studies show that the actions of IL-6 do not require PAR2. Our prior work also found a direct sensitizing action of IL-6 on dural afferents [28] providing a mechanistic basis for neuronal actions of IL-6 that are independent of immune cells.

## Conclusions

Numerous studies have been performed using GTN as a provocative model of migraine in both humans and animals. Despite these studies, mechanisms by which NO contribute to migraine are not known. Nonetheless, sensitization to NO seems to play an important role in the disorder. Here we show that both PAR2 activation and IL-6 signaling are capable of causing priming to an NO donor using distinct mechanisms. Better understanding of how these stimuli cause sensitization to NO may lead to new therapeutic discoveries. Therapeutics targeting IL-6 are currently being used in humans but the efficacy of these therapeutics in migraine has not been established. The findings shown here, combined with prior work from our group and others, strongly suggest that PAR2 should be explored a potential therapeutic target for migraine [23].

## Supplementary Information

**Additional file 1.**

## Data Availability

The datasets used and/or analysed during the current study are available from the corresponding author on reasonable request.

## Abbreviations

PAR2: Protease-activated receptor-2
NO: Nitric oxide
GTN: Glyceryl trinitrate
2AT: 2At-LIGRL-NH_2_
PAR2KO: PAR2 knockout
WT: Wild type
IL-6: Interleukin-6
ERK: Extracellular signal related kinase
MAPK: Mitogen activated protein kinase
