# Supplementary material for: PAR2 activation in the dura causes acute behavioral responses and priming to glyceryl trinitrate in a mouse migraine model
Source: J Headache Pain. 2023 Apr 19;24(1):42. doi: 10.1186/s10194-023-01574-5 (PMC10114383; doi:10.1186/s10194-023-01574-5)
Supplement: Supplementary file 1 — Additional file 1. [file 10194_2023_1574_MOESM1_ESM.docx]

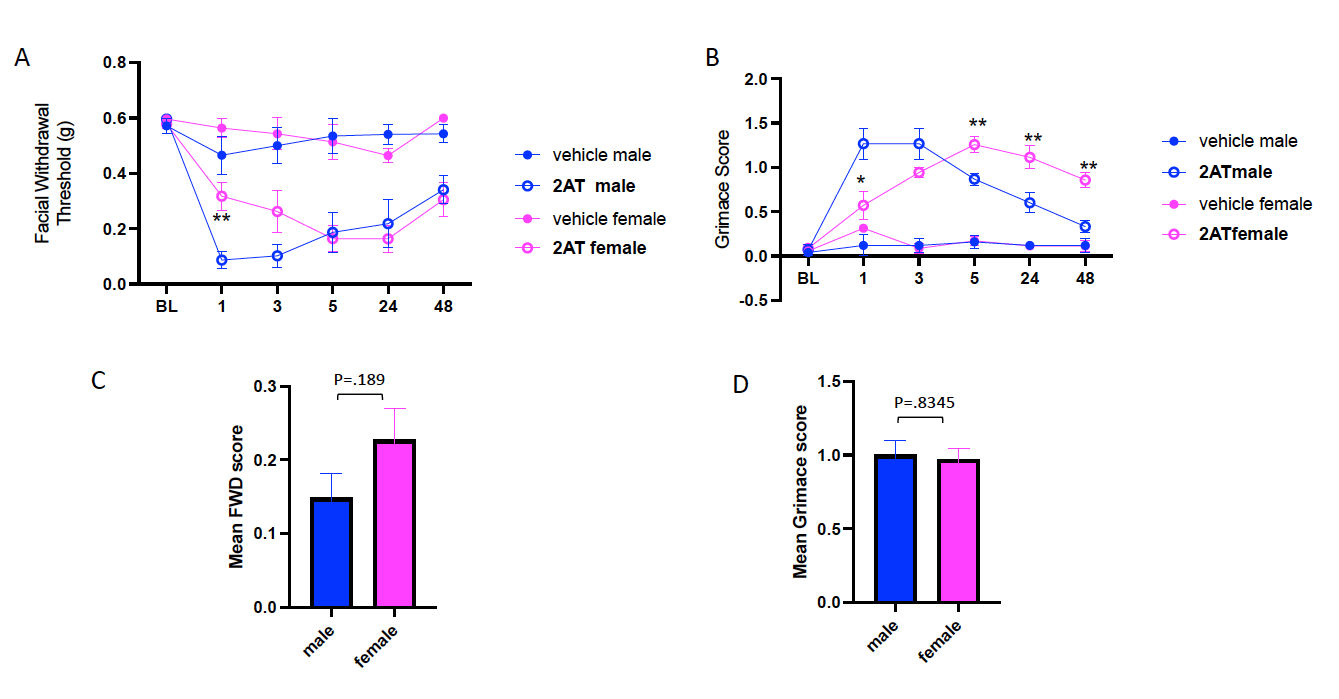


**Supplementary Figure 1.** Comparison of the time course between female and male mice following dural administration of 2AT. A. Facial withdrawal threshold following vehicle or 2AT injection on the dura of female (purple) and male (blue) mice. There is a significant difference in response to 2AT at 1 hour post-injection but not at any time point following 1 hour. B. Facial grimace scores following vehicle or 2AT injection on the dura of female (purple) and male (blue) mice. There is a significant difference at 1 hour and again at 5, 24, and 48 hours post-injection. C and D. Mean facial withdrawal threshold (represented as the mean of the gram filament response across time points from 1-48h) and grimace score over the entire time course showing no significant difference between sexes in the overall responses.


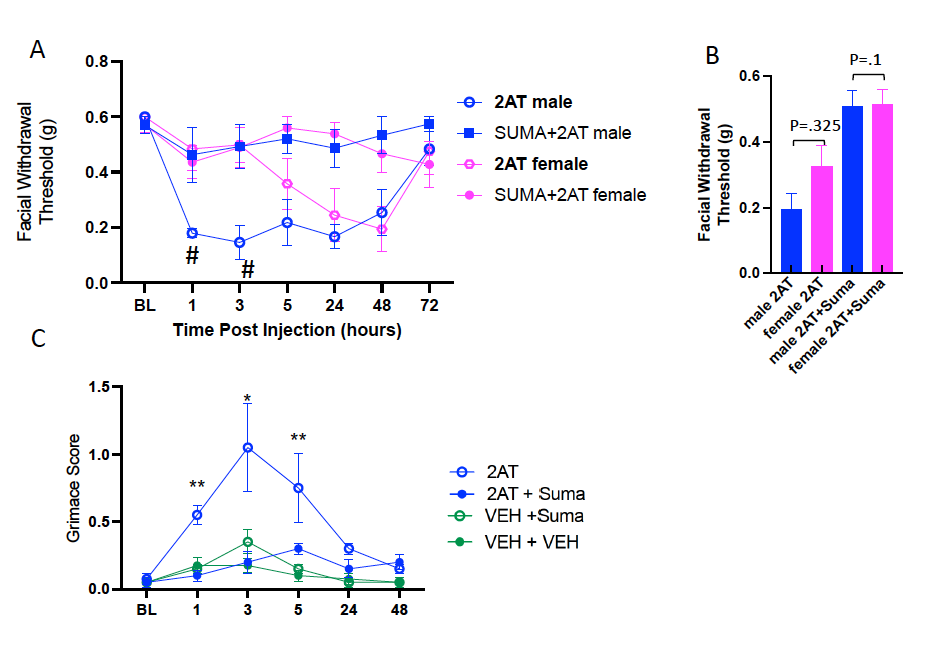


**Supplementary Figure 2.** Comparison of the 2AT response and effect of sumatriptan between female and male mice. A. Facial withdrawal threshold following 2AT or SUMA + 2AT injection on the dura of female (purple) and male (blue) mice. There is a significant difference in response to 2AT at 1 and 3 hours post-injection. B. Mean facial withdrawal threshold (represented as the mean of the gram filament response across 1-48h time points) showing no significant difference between sexes in the overall responses. C. Grimace scores in male mice following dural injection of 2AT or 2AT + sumatriptan (Suma). There is a significant increase in grimace scores from 1 to 3 hours post 2AT injection in the 2AT alone group only. There is no difference between the 2AT + sumatriptan group and the vehicle groups.


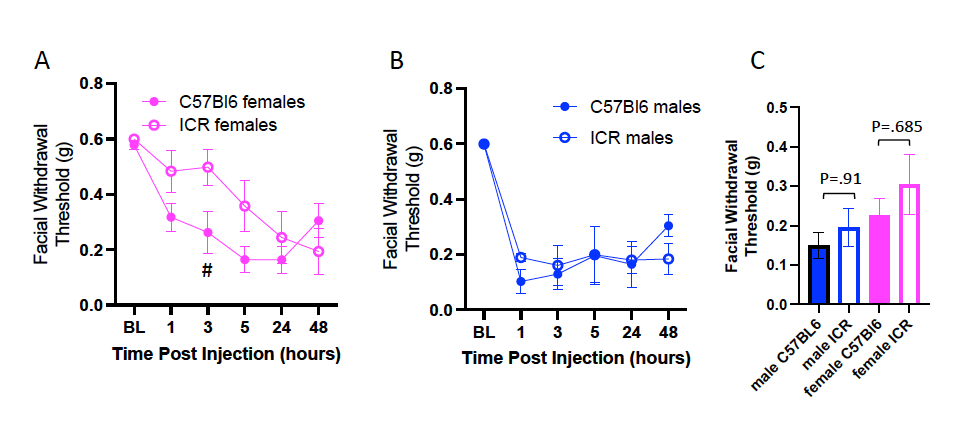


**Supplementary Figure 3.** Comparison in facial withdrawal threshold time course between C57BL6 and ICR mice following dural administration of 2AT. A is female and B is male mice. There is a significantly lower facial withdrawal threshold in C57BL6 mice at 3 hours post injection. There are no significant differences between mouse strains in male animals.
